# Redetermination of despujolsite, Ca_3_Mn^4+^(SO_4_)_2_(OH)_6_·3H_2_O

**DOI:** 10.1107/S1600536811030911

**Published:** 2011-08-06

**Authors:** Madison C. Barkley, Hexiong Yang, Stanley H. Evans, Robert T. Downs, Marcus J. Origlieri

**Affiliations:** a Department of Geosciences, University of Arizona, 1040 E. 4th Street, Tucson, Arizona 85721-0077, USA

## Abstract

The crystal structure of despujolsite [tricalcium manganese bis­(sulfate) hexahydroxide tri­hydrate], the Ca/Mn member of the fleischerite group, ideally Ca_3_Mn^4+^(SO_4_)_2_(OH)_6_·3H_2_O, was previously determined based on X-ray diffraction intensity data from photographs, without H-atom positions located [Gaudefroy *et al.* (1968 ▶). *Bull. Soc. Fr. Minéral. Crystallogr.* **91**, 43–50]. The current study redetermines the structure of despujolsite from a natural specimen, with all H atoms located and with higher precision. The structure of despujolsite is characterized by layers of CaO_8_ polyhedra (*m*.. symmetry) inter­connected by Mn(OH)_6_ octa­hedra (32. symmetry) and SO_4_ tetra­hedra (3.. symmetry) along [001]. The average Ca—O, Mn—O and S—O bond lengths are 2.489, 1.915, and 1.472 Å, respectively. There are two distinct hydrogen bonds that stabilize the structural set-up. This work represents the first description of hydrogen bonds in the fleischerite group of minerals.

## Related literature

For the previous determination of the despujolsite crystal structure, see: Gaudefroy *et al.* (1968 ▶). For background to fleischerite, see: Otto (1975 ▶). For the crystal structures of sulfate minerals with split O sites, see: Hill (1977 ▶); Jacobsen *et al.* (1998 ▶). For TLS (translation, libration, and screw motions) rigid-body analysis, see: Downs (2000 ▶). Parameters for bond-valence analysis were taken from Brese & O’Keeffe (1991 ▶).

## Experimental

### 

#### Crystal data

Ca_3_Mn(SO_4_)_2_(OH)_6_·3H_2_O
*M* *_r_* = 523.40
Hexagonal, 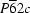

*a* = 8.5405 (5) Å
*c* = 10.8094 (9) Å
*V* = 682.81 (8) Å^3^
*Z* = 2
Mo *K*α radiation
μ = 2.49 mm^−1^
*T* = 293 K
0.07 × 0.06 × 0.04 mm

#### Data collection

Bruker APEXII CCD area-detector diffractometer
Absorption correction: multi-scan (*SADABS*; Sheldrick, 2008*a* ▶) *T*_min_ = 0.845, *T*_max_ = 0.907
6018 measured reflections
871 independent reflections
758 reflections with *I* > 2σ(*I*)
*R*_int_ = 0.051

#### Refinement

*R*[*F* ^2^ > 2σ(*F* ^2^)] = 0.024
*wR*(*F* ^2^) = 0.048
*S* = 1.06
871 reflections
49 parameters
All H-atom paramters refined
Δρ_max_ = 0.45 e Å^−3^
Δρ_min_ = −0.30 e Å^−3^
Absolute structure: Flack (1983 ▶), 305 Friedel pairs
Flack parameter: 0.0 (9)

### 

Data collection: *APEX2* (Bruker, 2003 ▶); cell refinement: *SAINT* (Bruker, 2005 ▶); data reduction: *SAINT*; program(s) used to solve structure: *SHELXS97* (Sheldrick, 2008*b* ▶); program(s) used to refine structure: *SHELXL97* (Sheldrick, 2008*b* ▶); molecular graphics: *XtalDraw* (Downs & Hall-Wallace, 2003 ▶); software used to prepare material for publication: *SHELXTL* (Sheldrick, 2008*b* ▶).

## Supplementary Material

Crystal structure: contains datablock(s) I, global. DOI: 10.1107/S1600536811030911/wm2518sup1.cif

Structure factors: contains datablock(s) I. DOI: 10.1107/S1600536811030911/wm2518Isup2.hkl

Additional supplementary materials:  crystallographic information; 3D view; checkCIF report

## Figures and Tables

**Table 1:** Selected bond lengths (Å)

Mn—O*H*3^i^	1.9149 (11)
Ca—O2^ii^	2.3465 (11)
Ca—O*H*3^i^	2.456 (5)
Ca—O*H*3^iii^	2.518 (5)
Ca—O*W*4	2.578 (9)
Ca—O*W*4^iv^	2.690 (9)
S—O2	1.4697 (11)
S—O1	1.4806 (18)

Symmetry codes: (i) 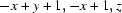; (ii) 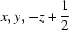; (iii) 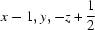; (iv) 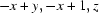.

**Table 2:** Hydrogen-bond geometry (Å, °)

*D*—H⋯*A*	*D*—H	H⋯*A*	*D*⋯*A*	*D*—H⋯*A*
O*H*3—H1⋯O2^v^	0.75 (2)	2.11 (2)	2.8193 (16)	158 (3)
O*W*4—H2⋯O1^vi^	0.77 (2)	2.10 (2)	2.7892 (18)	150 (3)

Symmetry codes: (v) 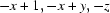; (vi) 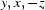.

**Fig. 1.**
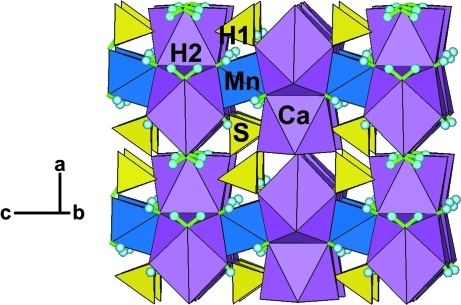
The crystal structure of despujolsite. Yellow tetrahedra represent SO4 groups. Blue octahedra and purple polyhedra represent Mn(OH)6 groups and Ca(O,OH,H2O)8 groups, respectively. Light blue spheres represent H1 and H2.

**Fig. 2.**
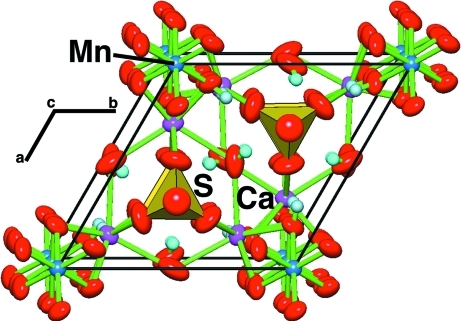
Atoms in despujolsite with corresponding ellipsoids at 99% probability. SO4 groups are treated as rigid bodies and shown as yellow tetrahedra. Blue, purple, and red ellipsoids represent Mn, Ca, and O atoms, respectively. Hydrogen atoms are shown as light-blue spheres.

**Fig. 3.**
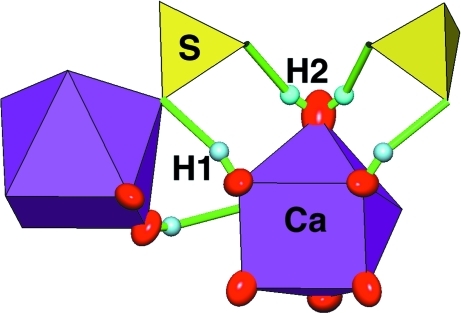
H-bonding interactions in despujolsite. Yellow tetrahedra represent SO4 groups. Purple polyhedra represent Ca(O,OH,H2O)8 groups. Red ellipsoids and blue spheres represent O and H atoms, respectively.

## supplementary crystallographic information

### Comment

Despujolsite, ideally Ca_3_Mn^4+^(SO_4_)_2_(OH)_6_^.^3H_2_O, is a member of the fleischerite group of minerals, which includes fleischerite, Pb_3_Ge(SO_4_)_2_(OH)_6_^.^3H_2_O, mallestigite, Pb_3_Sb(SO_4_)(AsO_4_)(OH)_6_^.^3H_2_O, and schauerteite, Ca_3_Ge^4+^(SO_4_)_2_(OH)_6_^.^3H_2_O. Thus far, only the structures of despujolsite and fleischerite (from a synthetic sample with the O1 site split) in this group have been determined (Gaudefroy *et al.*, 1968; Otto, 1975), both of which were based on X-ray diffraction intensity data measured from photographs, without H atom positions located. An *R*-factor of 0.162 was obtained for the structure model of despujolsite (Gaudefroy *et al.*, 1968). In our efforts to understand hydrogen bonding environments in general and the relationships in the hydrogen bonding schemes in the minerals of the fleischerite group in particular, we noted that the structural information of despujolsite needed to be improved.

The structure of despujolsite consists of layers of CaO_8_ polyhedra (*m*.. symmetry), interconnected by Mn(OH)_6_ octahedra (32. symmetry) and SO_4_ tetrahedra (3.. symmetry). The average Mn—O bond length is 1.915 Å (Table 1). Calculations of bond-valence sums using the parameters from Brese & O'Keeffe (1991) yield 3.86 (v.u.) for Mn, indicating that the assigned valence of 4+ for Mn is consistent with the structure. The average S—O bond length is 1.472 Å. Ca atoms are eight coordinated with 4 (OH)^-^ ions, 2 H_2_O molecules, and 2 O atoms. The average Ca—O bond length is 2.489 Å. There are two distinct hydrogen bonds: OH3—H1···O2 and OW4—H2··· O1 (Table 2).

The isostructural mineral fleischerite was previously modeled (Otto, 1975) with a split site for the O1 position. Similarly, studies of the sulfate mineral, barite BaSO_4_, refined the O atoms that lie on special positions with a split atom model (Hill, 1977). However, Hill notes that there are no significant improvements in the refinement with a split-site model over the symmetry-constrained model. Further structure refinement with significantly better data by Jacobsen *et al.* (1998) led to a TLS (translation, libration, and screw motions) rigid body analysis (Downs, 2000) of the sulfate group. The results indicated that the SO_4_ group behaves as a rigid body with significant translational and librational motions, demonstrating that the O atom sites are not split and that the large sizes of the displacement parameters are due entirely to thermal motion.

In contrast to the fleischerite study, our refinement of despujolsite did not indicate a split site. A TLS analysis of the displacement parameters indicate that the SO_4_ group in despujolsite behaves likewise as a rigid body with a translational amplitude of 0.72 Å and a large libration angle of 7.95°. The libration angle for the SO_4_ group in despujolsite, which is consistent with the 7°-8° range found in celestine, anglesite, and barite, indicates that the S–O bond lengths are ~0.009Å longer than their apparent values. If the libration angle is also large in fleischerite, then this may account for the effectiveness of splitting the O1 site, but our study indicates that the O1 site in fleischerite may not actually split.

### Experimental

The despujolsite specimen used in this study is from N'Chwaning III mine, Kalahari Manganese field, Northern Cape Province, South Africa and is in the collection of the RRUFF project (deposition No. R100208; http://rruff.info). The composition was determined with a CAMECA SX100 electron microprobe (http://rruff.info) on a single-crystal from the same parent sample as the crystal used for the collection of X-ray diffraction intensity data. Electron microprobe analysis (15 points) with a 15 K eV accelerating voltage, 20 nA beam current, and a 5 µm beam size yielded an empirical chemical formula Ca_3.45_Mn^4+^_0.86_(S_0.96_O_4_)_2_(OH)_6_^.^3H_2_O (based on 11 O atoms). Because despujolsite crystals are not stable under the electron beam, which has also been observed by Gaudefroy *et al.* (1968), the electron microprobe data were used only for the estimation of cation ratios. The actual composition was determined by a combination of microprobe and X-ray structural analyses. Details of the sample chemistry and structural formula calculations can be found on the RRUFF Project website (http://rruff.info/R100208).

### Refinement

The H atoms were located from difference Fourier maps and their positions were refined with isotropic displacement parameters. The final refinement assumed an ideal chemistry, as the overall effects of the trace amount of Fe and Si on the final structure results are negligible. The highest residual peak in the difference Fourier maps was located 0.66 Å from O1, and the deepest hole was located 0.40 Å from Mn.

### Crystal data

**Table:**

Ca_3_Mn(SO_4_)_2_(OH)_6_·3H_2_O	*D*_x_ = 2.546 Mg m^−^^3^
*M**_r_* = 523.40	Mo *K*α radiation, λ = 0.71073 Å
Hexagonal, *P*62*c*	Cell parameters from 1145 reflections
Hall symbol: P -6c -2c	θ = 2.8–32.0°
*a* = 8.5405 (5) Å	µ = 2.49 mm^−^^1^
*c* = 10.8094 (9) Å	*T* = 293 K
*V* = 682.81 (8) Å^3^	Euhedral, yellow
*Z* = 2	0.07 × 0.06 × 0.04 mm
*F*(000) = 530	

### Data collection

**Table:**

Bruker APEXII CCD area-detector diffractometer	871 independent reflections
Radiation source: fine-focus sealed tube	758 reflections with *I* > 2σ(*I*)
graphite	*R*_int_ = 0.051
φ and ω scans	θ_max_ = 32.6°, θ_min_ = 2.8°
Absorption correction: multi-scan (*SADABS*; Sheldrick, 2008a)	*h* = −12→11
*T*_min_ = 0.845, *T*_max_ = 0.907	*k* = −12→12
6018 measured reflections	*l* = −16→15

### Refinement

**Table:**

Refinement on *F*^2^	Hydrogen site location: difference Fourier map
Least-squares matrix: full	All H-atom parameters refined
*R*[*F*^2^ > 2σ(*F*^2^)] = 0.024	*w* = 1/[σ^2^(*F*_o_^2^) + (0.0113*P*)^2^ + 0.2266*P*] where *P* = (*F*_o_^2^ + 2*F*_c_^2^)/3
*wR*(*F*^2^) = 0.048	(Δ/σ)_max_ < 0.001
*S* = 1.06	Δρ_max_ = 0.45 e Å^−^^3^
871 reflections	Δρ_min_ = −0.30 e Å^−^^3^
49 parameters	Extinction correction: *SHELXL97* (Sheldrick, 2008), Fc^*^=kFc[1+0.001xFc^2^λ^3^/sin(2θ)]^-1/4^
0 restraints	Extinction coefficient: 0.0142 (11)
0 constraints	Absolute structure: Flack (1983), 305 Friedel pairs
Primary atom site location: structure-invariant direct methods	Flack parameter: 0.0 (9)
Secondary atom site location: difference Fourier map	

### Special details

**Table:**

Geometry. All e.s.d.'s (except the e.s.d. in the dihedral angle between two l.s. planes) are estimated using the full covariance matrix. The cell e.s.d.'s are taken into account individually in the estimation of e.s.d.'s in distances, angles and torsion angles; correlations between e.s.d.'s in cell parameters are only used when they are defined by crystal symmetry. An approximate (isotropic) treatment of cell e.s.d.'s is used for estimating e.s.d.'s involving l.s. planes.
Refinement. Refinement of *F*^2^ against ALL reflections. The weighted *R*-factor *wR* and goodness of fit *S* are based on *F*^2^, conventional *R*-factors *R* are based on *F*, with *F* set to zero for negative *F*^2^. The threshold expression of *F*^2^ > σ(*F*^2^) is used only for calculating *R*-factors(gt) *etc*. and is not relevant to the choice of reflections for refinement. *R*-factors based on *F*^2^ are statistically about twice as large as those based on *F*, and *R*- factors based on ALL data will be even larger.

### Fractional atomic coordinates and isotropic or equivalent isotropic displacement parameters (Å^2^)

**Table:**

	*x*	*y*	*z*	*U*_iso_*/*U*_eq_	
Mn	0.0000	0.0000	0.0000	0.00846 (12)	
Ca	0.1521 (3)	0.30348 (5)	0.2500	0.01085 (10)	
S	0.3333	0.6667	0.02544 (5)	0.00936 (12)	
O1	0.3333	0.6667	−0.11153 (15)	0.0167 (4)	
O2	0.2419 (10)	0.47842 (15)	0.06891 (10)	0.0188 (3)	
OH3	0.8945 (8)	0.0966 (8)	0.11070 (10)	0.0109 (3)	
OW4	0.5006 (12)	0.4853 (12)	0.2500	0.0171 (5)	
H1	0.836 (6)	0.125 (6)	0.076 (2)	0.026 (8)*	
H2	0.521 (9)	0.445 (9)	0.193 (2)	0.046 (10)*	

### Atomic displacement parameters (Å^2^)

**Table:**

	*U*^11^	*U*^22^	*U*^33^	*U*^12^	*U*^13^	*U*^23^
Mn	0.00919 (15)	0.00919 (15)	0.0070 (2)	0.00460 (8)	0.000	0.000
Ca	0.0137 (10)	0.00918 (19)	0.00980 (16)	0.0058 (10)	0.000	0.000
S	0.01001 (16)	0.01001 (16)	0.0081 (2)	0.00501 (8)	0.000	0.000
O1	0.0213 (6)	0.0213 (6)	0.0075 (7)	0.0107 (3)	0.000	0.000
O2	0.027 (3)	0.0106 (5)	0.0170 (5)	0.008 (2)	0.004 (3)	0.0033 (4)
OH3	0.0084 (15)	0.0148 (19)	0.0107 (4)	0.0067 (6)	−0.0006 (16)	−0.0004 (16)
OW4	0.019 (2)	0.023 (2)	0.0136 (6)	0.0138 (12)	0.000	0.000

### Geometric parameters (Å, °)

**Table:**

Mn—OH3^i^	1.9149 (11)	Ca—OH3^viii^	2.456 (5)
Mn—OH3^ii^	1.9149 (11)	Ca—OH3^iii^	2.518 (5)
Mn—OH3^iii^	1.9149 (11)	Ca—OH3^ix^	2.518 (5)
Mn—OH3^iv^	1.9149 (11)	Ca—OW4	2.578 (9)
Mn—OH3^v^	1.9149 (11)	Ca—OW4^x^	2.690 (9)
Mn—OH3^vi^	1.9149 (11)	S—O2^x^	1.4697 (11)
Ca—O2^vii^	2.3465 (11)	S—O2^xi^	1.4697 (11)
Ca—O2	2.3465 (11)	S—O2	1.4697 (11)
Ca—OH3^i^	2.456 (5)	S—O1	1.4806 (18)
			
OH3^i^—Mn—OH3^ii^	177.7 (4)	OH3^ii^—Mn—OH3^vi^	85.08 (5)
OH3^i^—Mn—OH3^iii^	85.08 (5)	OH3^iii^—Mn—OH3^vi^	96.5 (3)
OH3^ii^—Mn—OH3^iii^	93.4 (3)	OH3^iv^—Mn—OH3^vi^	177.7 (4)
OH3^i^—Mn—OH3^iv^	85.08 (5)	OH3^v^—Mn—OH3^vi^	85.08 (5)
OH3^ii^—Mn—OH3^iv^	96.5 (3)	O2^x^—S—O2^xi^	110.28 (5)
OH3^iii^—Mn—OH3^iv^	85.08 (5)	O2^x^—S—O2	110.28 (5)
OH3^i^—Mn—OH3^v^	96.5 (3)	O2^xi^—S—O2	110.28 (5)
OH3^ii^—Mn—OH3^v^	85.08 (5)	O2^x^—S—O1	108.65 (5)
OH3^iii^—Mn—OH3^v^	177.7 (4)	O2^xi^—S—O1	108.65 (5)
OH3^iv^—Mn—OH3^v^	93.4 (3)	O2—S—O1	108.65 (5)
OH3^i^—Mn—OH3^vi^	93.4 (3)		

Symmetry codes: (i) −*x*+*y*+1, −*x*+1, *z*; (ii) *x*−*y*−1, −*y*, −*z*; (iii) *x*−1, *y*, *z*; (iv) −*y*, *x*−*y*−1, *z*; (v) *y*, *x*−1, −*z*; (vi) −*x*+1, −*x*+*y*+1, −*z*; (vii) *x*, *y*, −*z*+1/2; (viii) −*x*+*y*+1, −*x*+1, −*z*+1/2; (ix) *x*−1, *y*, −*z*+1/2; (x) −*x*+*y*, −*x*+1, *z*; (xi) −*y*+1, *x*−*y*+1, *z*.

### Hydrogen-bond geometry (Å, °)

**Table:**

*D*—H···*A*	*D*—H	H···*A*	*D*···*A*	*D*—H···*A*
OH3—H1···O2^xii^	0.75 (2)	2.11 (2)	2.8193 (16)	158 (3)
OW4—H2···O1^xiii^	0.77 (2)	2.10 (2)	2.7892 (18)	150 (3)

Symmetry codes: (xii) −*x*+1, −*x*+*y*, −*z*; (xiii) *y*, *x*, −*z*.
